# Phenotypic divergence among threespine stickleback that differ in nuptial coloration

**DOI:** 10.1002/ece3.6105

**Published:** 2020-02-18

**Authors:** Clara S. Jenck, Whitley R. Lehto, Brian T. Ketterman, Lukas F. Sloan, Aaron N. Sexton, Robin M. Tinghitella

**Affiliations:** ^1^ Department of Biological Sciences University of Denver Denver CO USA; ^2^ Department of Integrative Biology Michigan State University East Lansing MI USA; ^3^ Duke University School of Medicine Durham NC USA; ^4^ Department of Biology University of Louisville Louisville KY USA

**Keywords:** color morph, *Gasterosteus aculeatus*, phenotypic divergence, threespine stickleback

## Abstract

By studying systems in their earliest stages of differentiation, we can learn about the evolutionary forces acting within and among populations and how those forces could contribute to reproductive isolation. Such an understanding would help us to better discern and predict how selection leads to the maintenance of multiple morphs within a species, rather than speciation. The postglacial adaptive radiation of the threespine stickleback (*Gasterosteus aculeatus*) is one of the best‐studied cases of evolutionary diversification and rapid, repeated speciation. Following deglaciation, marine stickleback have continually invaded freshwater habitats across the northern hemisphere and established resident populations that diverged innumerable times from their oceanic ancestors. Independent freshwater colonization events have yielded broadly parallel patterns of morphological differences in freshwater and marine stickleback. However, there is also much phenotypic diversity within and among freshwater populations. We studied a lesser‐known freshwater “species pair” found in southwest Washington, where male stickleback in numerous locations have lost the ancestral red sexual signal and instead develop black nuptial coloration. We measured phenotypic variation in a suite of traits across sites where red and black stickleback do not overlap in distribution and at one site where they historically co‐occurred. We found substantial phenotypic divergence between red and black morphs in noncolor traits including shape and lateral plating, and additionally find evidence that supports the hypothesis of sensory drive as the mechanism responsible for the evolutionary switch in color from red to black. A newly described third “mixed” morph in Connor Creek, Washington, differs in head shape and size from the red and black morphs, and we suggest that their characteristics are most consistent with hybridization between anadromous and freshwater stickleback. These results lay the foundation for future investigation of the underlying genetic basis of this phenotypic divergence as well as the evolutionary processes that may drive, maintain, or limit divergence among morphs.

## Graphical abstract

Unveiling when or how traits are selected for or against is key to understanding the patterns of phenotypic variation observed in natural populations, especially when multiple morphs are maintained. We measured a comprehensive suite of phenotypic traits in two different freshwater threespine stickleback color morphs, red‐throated and black‐bodied. We found extensive divergence in nuptial color, shape, and body armor between red and black stickleback, and also form a hypothesis for the existence of a possible third, hybrid morph. This is the first in‐depth investigation of variation in morphological traits in populations of red and black stickleback.

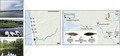

## 1 INTRODUCTION

Much of the historical work on the origin and maintenance of biodiversity has relied heavily on the characterization of phenotypic variation as a basis for inferring the existence and trajectory of evolutionary change (Darwin, 1859; Endler, 1980; Grant & Grant, 2002; Losos, 1990; Schluter, 2000; Wallace, 1871). The substantial variation in traits we observe among taxa supports the hypothesis that divergent selection can drive reproductive isolation, which builds as a result of adaptation to contrasting selection regimes imposed by different environments (Schluter, 2001). Both natural selection and sexual selection are important evolutionary forces that can generate and shape phenotypes and also have roles to play in the generation of biodiversity (speciation; Ritchie, 2007; Safran et al., 2013; Servedio & Boughman, 2017).

Recent work has emphasized how natural selection and sexual selection work jointly to drive evolutionary change, divergence, and even speciation (Safran et al., 2013). Divergent natural selection among populations can arise because of differences in factors including habitat, resources, climate, and predation (Schluter, 2001). In three lizard species that inhabit the White Sand dunes in New Mexico, for instance, cryptic coloration has rapidly evolved and is selectively maintained by predation, relative to their background environment (Rosenblum et al., 2010). These environmental factors can also affect sexually selected traits. For example, interactions with eavesdropping predators and parasites (reviewed in Zuk & Kolluru, 1998), interspecific (reviewed in Gröning & Hochkirch, 2008) and intraspecific competing signalers (reviewed in Tinghitella, Lackey, et al., 2018), and transmittance properties of the environment (Boughman, 2002; Endler, 1992; Seehausen et al., 2008) place sexually selected traits under conflicting selection that shapes phenotypic and genetic variation within and among populations. Thus, natural selection can impose a cost on conspicuous sexual displays, such as in the Pacific field cricket (*Teleogryllus oceanicus*) where male calling song also attracts parasitoids (Zuk et al., 2006) and in guppies (*Poecilia reticulata*) where environmental conditions affect the transmittance of light and perception of colorful sexual signals (Endler, 1991; Gamble, Lindholm, Endler, & Brooks, 2003). Here, we measure phenotypic change in a suite of traits across several populations of fish that have undergone recent divergence in their sexual signals, likely as a consequence of habitat variation.

Species that have diversified over relatively short time scales and that are distributed across landscapes with varied environmental characteristics likely to generate divergent selection shed important light on the evolutionary processes underlying phenotypic change. The threespine stickleback (*Gasterosteus aculeatus*) is one such model study system. These fish episodically colonized freshwater habitats from marine environments following glacial retreat at the end of the Pleistocene epoch less than 12,000 years ago (McPhail, 1994). In many cases, the resulting freshwater populations have diverged phenotypically (reviewed in McKinnon & Rundle, 2002) and genetically (Colosimo et al., 2005; Cresko et al., 2004; Currey, Bassham, & Cresko, 2019; Hohenlohe et al., 2010; Jones, Chan, et al., 2012) from marine ancestors in parallel ways, offering natural, replicated, and independent evolutionary experiments. Upon colonizing freshwater habitats, stickleback experience selection that leads to divergence in color, shape, size, salinity tolerance, and foraging behavior and morphology. Stickleback populations have also undergone divergence in the presence and number of lateral bony plates, a trait that has quite famously evolved repeatedly and predictably in response to freshwater–marine differences such as predation and salinity (Bell, 2001; Marchinko & Schluter, 2007; Reimchen & Nosil, 2004). Typically, marine fish are larger and have fully plated bodies whereas stream‐dwelling freshwater stickleback are smaller and tend to have low or partial plating (Hagen & Gilbertson, 1973; Bell & Foster, 1994).

In some cases, sexually selected traits have also undergone rapid evolutionary change in freshwater stickleback populations. Like ancestral marine stickleback, male stickleback from most derived freshwater populations display a bright red throat during the breeding season (hereafter referred to as red stickleback; Hagen & Moodie, 1979; McPhail, 1969; Semler, 1971). However, in several locations along the Pacific coast of North America, males have lost their iconic mating signal and instead have full‐body black breeding coloration (hereafter referred to as black stickleback; Boughman, 2001; McPhail, 1969; Reimchen, 1989; Semler, 1971). This red and black stickleback system is often considered a “species pair” in the literature (McKinnon & Rundle, 2002); we hereafter refer to them as color morphs. The prevailing explanation for this evolutionary switch is sensory drive, the process by which sexual signals shift to improve transmittance in their environment (Boughman, 2002; Endler, 1992). Red stickleback are often found in habitats with relatively clear water whereas black stickleback are found in red‐shifted, tannin‐rich waters, making males of each color morph highly contrasted and more visible to the drab females in their respective environments (Boughman, 2001; Reimchen, 1989; Scott, 2001). Boughman (2001) shows that in red (limnetic—relatively clear water) and black (benthic—relatively red‐shifted water) British Columbian stickleback from Paxton Lake and Enos Lake, the extent of divergence in male color and female preference for male color is correlated with the extent of reproductive isolation among populations, supporting a role for sensory drive in speciation.

Recent work in red and black stickleback from Washington State similarly supports a role for sexual selection in the divergence of red and black stickleback, albeit through changes in male competition behavior, rather than female preferences (Tinghitella et al., 2015; Tinghitella, Lehto, et al., 2018). In simulated secondary contact in the laboratory, females from populations containing only red or only black males retain their ancestral preference for the red mating signal (McKinnon, 1995) and prefer to interact with red males (Tinghitella et al., 2015). Though there is no evidence of assortative mating, male competition for territories, which occurs prior to female mate choice in the breeding season, may be an important isolating mechanism in this system; in Washington, black males bias their aggression toward red males, so red males receive more aggression overall than black males. Such a pattern may contribute to habitat and reproductive isolation between the two color morphs (Tinghitella et al., 2015).

Different traits are frequently correlated and genetically linked to one another, so the recent and rapid changes in freshwater stickleback body color may be associated with changes in a suite of traits that are associated with reproductive isolation. In Enos Lake, for instance, body shape is correlated with male nuptial color such that deeper bodied fish have redder throats (Malek, Boughman, Dworkin, & Peichel, 2012), suggesting genetic linkage of the two. Additionally, several studies have suggested a role for body size and shape in the adaptive divergence of stickleback and as a driver of prezygotic reproductive isolation through size‐assortative mating (Head, Kozak, & Boughman, 2013; McPhail, 1977; Nagel & Schluter, 2006). In this study, we measure a comprehensive suite of phenotypic traits that have evolved in parallel as stickleback colonized freshwater habitats(McKinnon & Rundle, 2002) including nuptial color, shape, size, and body armor in Washington populations of red and black stickleback.

Unveiling when or how traits undergo selection is key to understanding the patterns of phenotypic variation observed in natural populations. Additionally, assessing variation in locations where multiple morphscoexist and possibly interbreed can offer even more insight into the processes that maintain biodiversity (Gray & McKinnon, 2007; Hoekstra, Drumm, & Nachman, 2004; Roulin, 2004; Rueffler et al., 2006; Schluter, 2000). In pioneering work, McPhail (1969), and Hagen and Moodie (1979), found a region in southwest Washington, Connor Creek, where both red and black stickleback were found with overlapping breeding areas and seasons. Our own surveys in 2018 revealed a site with only black fish plus locations where males had apparent continuous variation in color that prevented us from characterizing fish as clearly red or black. If red and black stickleback interbreed within Connor Creek, we may find a phenotypic cline indicating the presence of a hybrid zone or localized adaptation to an environmental gradient (Endler, 1977). Given the variation in nuptial color between morphs, the correlated evolution of shape and color (Malek et al., 2012), and the parallel evolutionary loss of plating in freshwater stickleback across the northern hemisphere, we expect red and black color morphs to differ in body shape, size, and plating, in addition to color. We surveyed phenotypic divergence of stickleback across six sites where red and black fish are allopatric (nonoverlapping in distribution) and also take a finer‐scale approach by examining the phenotypic divergence of color morphs where they historically co‐occurred in a single location. To our knowledge, this is the first in‐depth investigation of variation in morphological traits (aside from coloration) in WA populations of red and black stickleback.

## 2 METHODS

### 2.1 Sample collection

**Figure 1:**
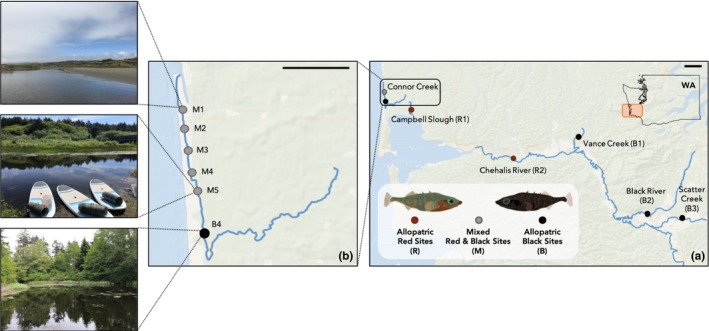
(a) Washington sites used in morphological analyses. Sites where we collect red stickleback are denoted with “R” and red‐colored points, sites where we collect black stickleback are denoted with “B” and black‐colored points, and sites where we collect mixed stickleback are denoted with “M” (for mixed) and gray‐colored points. (b) Connor Creek collection sites mirror those of McPhail (1969). Mixed stickleback were collected from locations M1–M5, whereas only black stickleback were collected from site B4. The images depict the drastic habitat transition from areas with high vegetation and deep water (B4) to sand dunes and shallow water (M1) as the creek approaches the Pacific Ocean. Black bars in the top right corner of each panel correspond to a distance of five kilometers

We collected sexually mature, adult stickleback from streams and rivers of southwest Washington, United States, and transferred them to the University of Denver during the summers of 2013–2015 (Table 1; Figure 1a). Fish with red nuptial coloration were collected from two sites (Campbell Slough (R1) and Chehalis River (R2)) where black fish are not found, and fish with black nuptial coloration were collected from four sites (Vance Creek (B1), Black River (B2), Scatter Creek (B3), and Connor Creek (B4)) where red fish are not found. In summer 2018, we collected stickleback along a 3.5 km transect in Connor Creek, where both color morphs have historically coexisted (Hagen & Moodie, 1979; McPhail, 1969). To parallel the sampling first done by McPhail (1969), we sampled five locations by paddleboarding along the transect, trapping at approximately 0.9‐kilometer intervals, beginning near the mouth of the creek (M1) and moving further inland toward our 2015 Connor Creek sampling site where only black fish are found (B4; Figure 1b). While we sampled five locations along the transect, fish did not appear to differ in color between locations. Thus, for the purpose of our phenotypic analyses, we hereafter refer to these five Connor Creek locations within our finer‐scale approach as one collective “mixed” site, M1–M5. We collected stickleback using nonbaited, galvanized steel mesh minnow traps. All methods were approved by the University of Denver's IACUC (protocol 883302–9), and fish were collected under Washington Department of Fish and Wildlife Scientific Collection permits 16–208, 17–134, and 18–173.

**Table 1:** Collection site details including site ID, color morph found at each, GPS coordinates, and stickleback and water sample collection year(s). From each collection site, the number of males and females included in shape and size analyses, the number of only males included in the color analysis, and the number of males and females total included in the plating analysis. Sites with only red fish are denoted with “R,” sites with only black fish are denoted with “B,” and sites with mixed fish are denoted with “M”

Site	Color morph	GPS coordinates	Stickleback collection year(s)	Water sample collection year	Shape & size		Color	Plating
					Males	Females	Males	Males & Females
Campbell Slough (R1)	Red	47°2ʹ40ʺN	May 2013, 14, 15	2013	42	31	33	42
		124°3ʹ33ʺ W						
Chehalis River (R2)	Red	46°56ʹ22ʺN	May 2013, 14, 15	2013	57	49	21	41
		123°18ʹ46ʺW						
Vance Creek (B1)	Black	46°59ʹ48ʺN	May 2013, 14, 15	2013	34	12	26	42
		123°24ʹ43ʺW						
Black River (B2)	Black	46°49ʹ45ʺN	May 2013, 14, 15	2013	41	46	23	40
		123°8ʹ1ʺW						
Scatter Creek (B3)	Black	46°49ʹ20ʺN	May 2013, 14, 15	2013	40	22	22	42
		123°3ʹ11ʺW						
Connor Creek (B4)	Black	47°4ʹ11ʺN	May 2013, 14, 15	2018	35	28	24	41
		124°10ʹ5ʺW						
Connor Creek (M1)	Mixed	47°6ʹ55ʺN	May 2018	2018	5	–	5	30
		124°10ʹ52ʺW						
Connor Creek (M2)	Mixed	47°6ʹ26ʺN	May 2018	2018	7	–	7	32
		124°10ʹ45ʺW						
Connor Creek (M3)	Mixed	47°5ʹ57ʺN	May 2018	2018	6	–	6	26
		124°10ʹ39ʺW						
Connor Creek (M4)	Mixed	47°5ʹ29ʺN	May 2018	2018	3	–	3	18
		124°10ʹ30ʺW						
Connor Creek (M5)	Mixed	47°5ʹ12ʺN	May 2018	2018	2	–	2	46
		124°10ʹ20ʺW						

### 2.2 Colorimetric water collection

Sensory drive is the prevailing explanation for the evolutionary switch from red nuptial coloration to black nuptial coloration and may be important at our sampling sites (Boughman, 2001; Reimchen, 1989; Scott, 2001). To test for an association between water color and stickleback color morphs, we collected three to five water samples from each site, as well as the five locations along the Connor Creek transect, and returned them to the laboratory for colorimetric analyses (Table 1). We measured the transmittance of light through each water sample using a spectrophotometer at wavelengths of 340, 405, 490, 550, 595, and 650 nm, calibrating with distilled water (100% transmittance) before each new sample following Scott (2001).

### 2.3 Phenotypic data collection

All stickleback from the 2013, 2014, 2015, and 2018 collections were housed in the laboratory and maintained in visually isolated single‐sex 110‐L holding tanks (77 × 32 × 48 cm), separated by population at densities of ~ 30 fish per tank. Fish tanks were placed in a room with controlled lighting and temperature (set to a 15:9 light:dark schedule and 17°C) at the beginning of the season. Laboratory conditions tracked those occurring in southwest Washington to mimic breeding conditions throughout the remainder of the season. Lights in the room are broad‐spectrum Sylvania Octron Eco 5000‐K fluorescent lights. We fed fish a diet of bloodworms (*Chironomus* spp.) and brine shrimp (*Artemia* spp.) daily. After transportation to the laboratory, we allowed the fish acclimate for two weeks before undergoing any phenotyping.

To conduct no‐choice mating trials (not reported on here), we moved males who showed nuptial color and mating behavior into single‐male, 284‐L nesting tanks (123 × 47 × 54 cm) containing all of the items necessary to begin nesting (a tray of sand covered by one half of a flower pot, an artificial plant, and nesting material). When males had completed nest building, a single gravid female—identified on the basis of a distended abdomen and the presence of ripe eggs—was released into the male's tank for a mating trial. The photographs we analyzed in this study were taken immediately following the completion of the 20‐min trial (or when the female entered the male's nest). Individuals were photographed using a digital camera (Canon PowerShot G15) under standardized lighting conditions (four evenly spaced xenon 20 W light bulbs) inside of a photo box that blocked ambient light. The camera was placed at a fixed distance from a neutral background, which was used for white‐balance adjustment. We positioned fish on their right side, unanesthetized, below a millimeter ruler. The photographing process takes less than 30 s to minimize any changes in the expression of male nuptial color. Males and females were individually marked with colored elastomer tags and were not used in more than three mating trials. Each male was always paired with a different female, and all trials were conducted during the breeding season (June to September) when males displayed nuptial coloration. Stickleback collected in a given year (e.g., May 2013 collection) were only used in behavioral trials and phenotyping during that breeding season (e.g., June to September 2013); as these stickleback are annual, though there were multiple seasons of collection, fish were not used across years.

We measured four morphological traits on males and females from 11 sites total (sample sizes are found in Table 1). We used the photographs from mating trials to quantify color, shape, and size. When several photographs of the same fish existed, we used a random number generator to determine which image to analyze, ensuring that photographs of fish taken at particular time points in the breeding season were not selected preferentially. All shape, size, and color data were collected using FIJI (ImageJ; Schindelin et al., 2012). For each photograph, we set a scale factor using the ruler above the fish, cropped the image to only include the individual, and deleted the caudal fin, as it does not always lay flat in photographs. This image was then used for the assessment of color, shape, and size.

### 2.4 Male color

**Figure 2:**
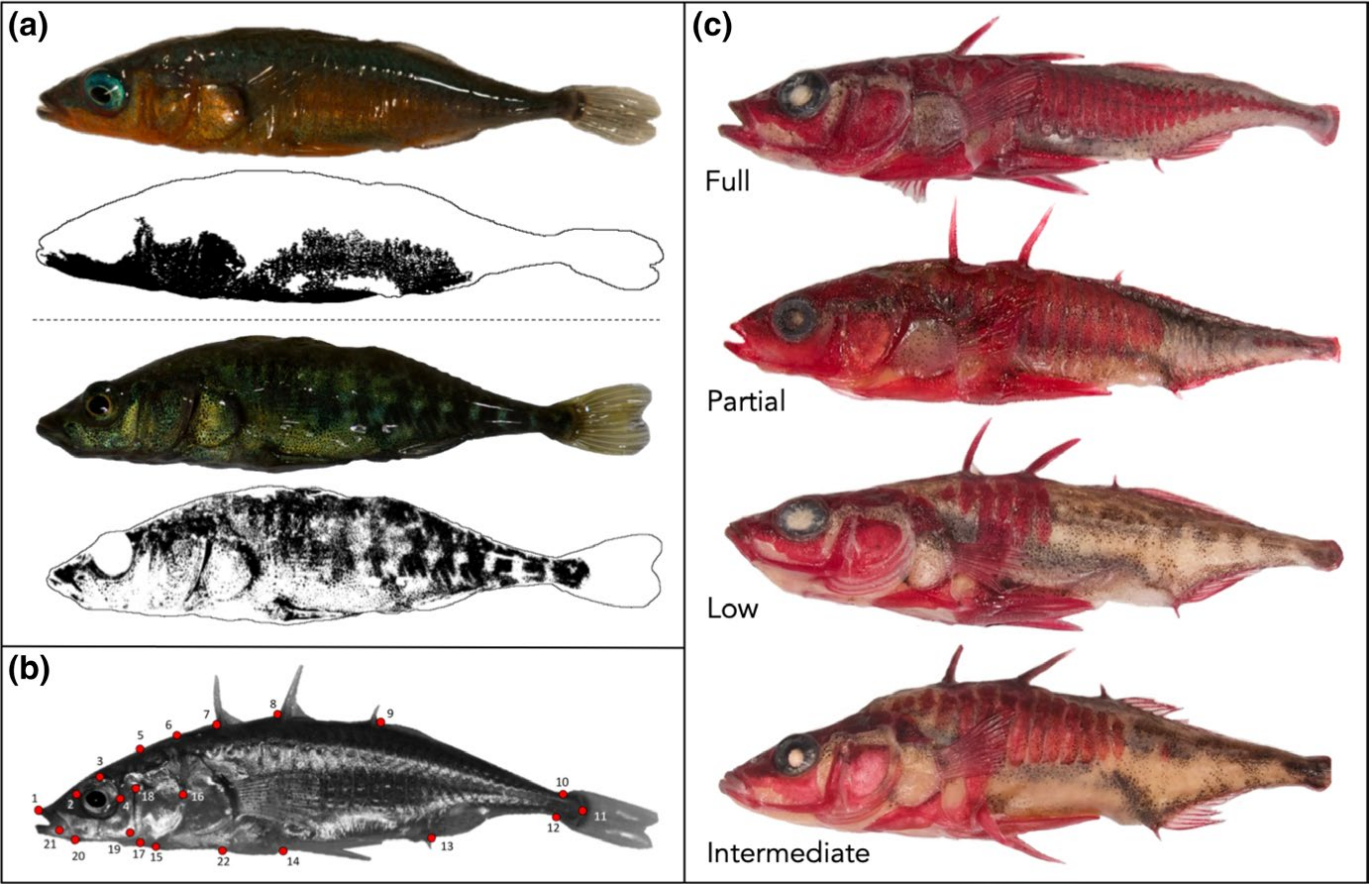
Color and morphological traits measured. (a; top) A red male with quantification of red coloration and (a; bottom) a black male with quantification of black coloration. (b) A male displaying the 22 landmarks used to conduct shape and size analyses. (c) Four individuals representing each category of plating

Because female stickleback at sites containing both red and black male color morphs are drab, we only analyzed color in 172 males. All males expressed nuptial coloration at the time of photographing. We measured red and black coloration as a proportion of total body area on the same image of each fish, after additionally removing the area of the eyeball from the image (Figure 2a). First, we selected red coloration using the Threshold Color plugin within FIJI, capturing all areas ranging from yellow to red to purple (Y = 32–255, U = 0–143, V = 141–255; following Wong et al., 2007 and Tinghitella, Lehto, et al., 2018). To select black coloration, we converted the image to 8‐bit grayscale and used the Threshold Color plugin (Y = 0–25, U = 0–255, V = 0–255), with which the entire area of the selected pixels was measured. We determined total body area using the SIOX (Simple Interactive Object Extraction) segmentation.

### 2.5 Body shape and size

We carried out morphometric analyses to quantify shape of 460 male and female stickleback by placing 22 landmarks on each image and collecting their X–Y coordinates (Figure 2b). These landmarks have been previously established to best capture shape variation in stickleback (Albert et al., 2007; Cooper, Gilman, & Boughman, 2011; Head et al., 2013; Malek et al., 2012; Taylor et al., 2006; Walker & Bell, 2000). We then quantified overall body size of males using centroid size as our measure (the square root of the sum of squared distances of all landmarks from their centroid; Head et al., 2013; Wund et al., 2012). Our sampling regime did not include photos of females from the Connor Creek mixed sites (M1–M5) so they were excluded from shape and size analyses.

### 2.6 Lateral plating

Following their natural death in the laboratory, we stored fish by collection site in jars containing 90% ethanol. To quantify lateral plating across morphs, we stained fish with Alizarin red following standardized methods in Cresko et al. (2004). We counted lateral body plates on both sides of 400 fish from 11 sites and additionally categorized each individual as having full, partial, intermediate, or low plating (Figure 2c). Following Bell (1982), we considered fish to be fully plated if they had a continuous row of plates from the head to the caudal peduncle, low plated if plating was strictly restricted to the abdominal region, and partially plated if they had both abdominal plating and a row of plating near the caudal peduncle that were separated by a gap with no plating. During the staining process, we discovered fish from our most recent collection in Connor Creek that could not fit into any of these categories. Similar to Bell, Aguirre, and Buck (2004), these atypical individuals were denoted as “intermediate,” as they had a row of plates that extended beyond the abdominal region but did not have a row of plating near the caudal peduncle (i.e., not low or partial plating).

### 2.7 Statistical analyses

**Figure 3:**
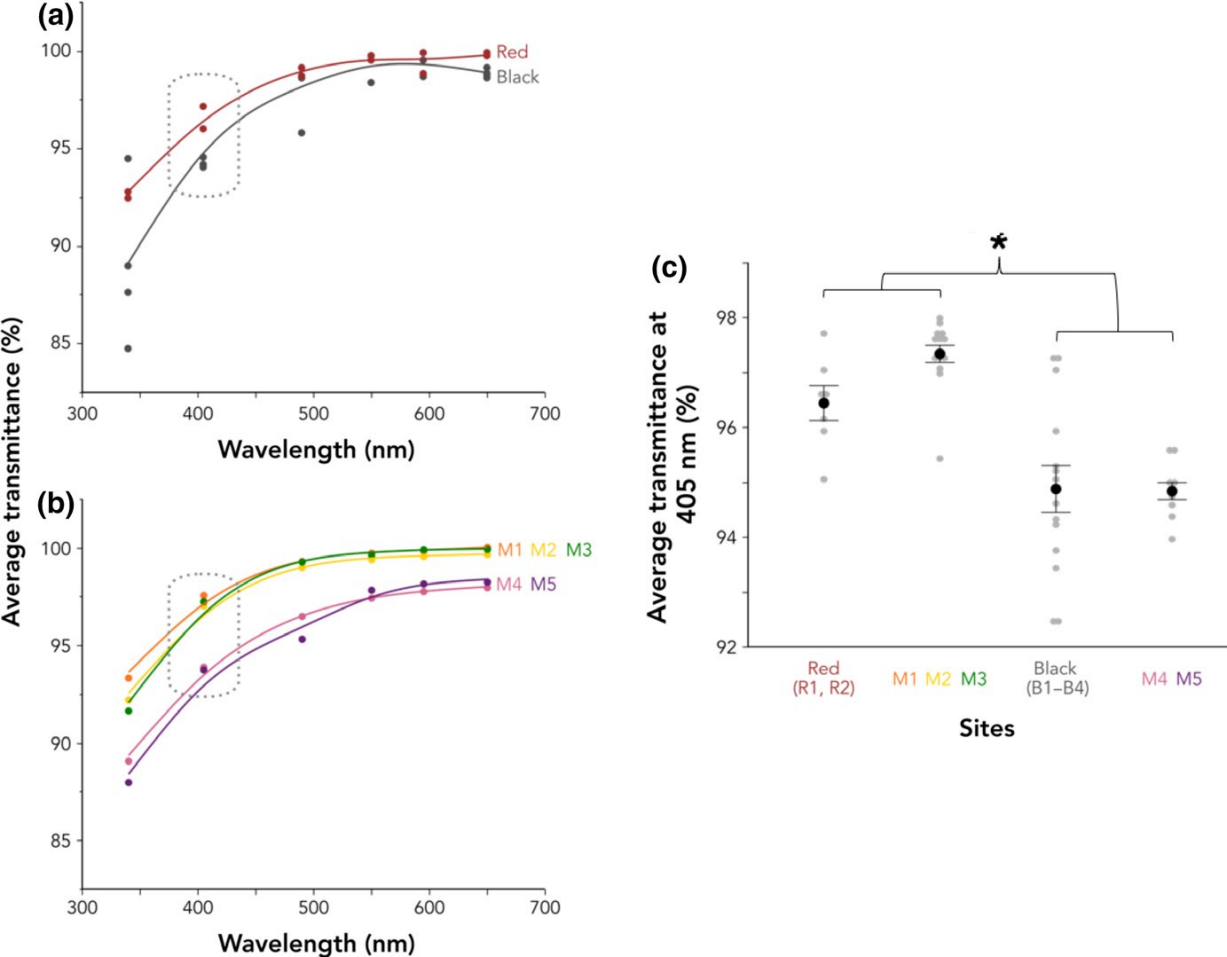
(a) Average transmittance of light through water samples from red and black collection sites and (b) from the five collection locations only along the Connor Creek transect at a range of wavelengths. Each data point represents the mean of three to five replicate samples and dotted gray boxes indicate transmittance at 405 nm. (c) At 405 nm, transmittance of light does not differ between red sites and the three Connor Creek locations closest to the ocean (“coastal mixed”: M1, M2, and M3), or between black sites and the two Connor Creek locations furthest inland (“inland mixed”: M4 and M5). Transmittance differs in all comparisons within these two groupings. Gray points represent raw values, black points and bars represent the least square means from the analysis plus or minus *SE*, and the asterisk indicates a significant difference using Tukey's HSD (*p* ≤ .05)

Following Reimchen (1989) and Scott (2001), we used average transmittance at 405 nm as our standard measure of water color, as tannin staining is best indicated by low transmittance at shorter wavelengths and shorter wavelengths are the most variable among our collection sites. We conducted a one‐way ANOVA to compare the effect of sampling site on the transmittance of light through water samples followed by Tukey's HSD (with alpha = .05) to find pairwise comparisons. After visualizing the distribution of transmittance at our collection sites across a range of wavelengths (Figure 3a and b), we compared the average transmittance of light at 405 nm among sites we categorize as red (R1 and R2), sites we categorize as black (B1–B4), the three mixed locations closest to the ocean (M1–M3), and the two mixed locations furthest inland (M4 and M5), as there was apparent distinction between these four groups across wavelengths.

To quantitatively determine whether what we refer to as red and black morphs differ in coloration, we first performed a regression of black area on red area to obtain residuals for every individual, allowing us to represent color as a single variable. Increasingly negative residuals indicate redder fish whereas increasingly positive residuals indicate blacker fish. We then used one REML linear mixed model with residual color as the outcome variable, categorical color morph (red, black, mixed) as a fixed effect, and site as a random effect to assess differences in male color among morphs, and another with site as a fixed effect to assess differences in male color among sites. We used Tukey's HSD (with alpha = .05) to find pairwise comparisons.

We conducted the analysis of morphometric data in MorphoJ version 2.0 (Klingenberg, 2011). The landmark X–Y coordinates were imported into the program and, following Lackey and Boughman (2013), we used the Procrustes transformation to center, scale, and align the coordinates, allowing for comparisons of each landmark across images by controlling for the relative size and position of each individual. We used methods established by Drake and Klingenberg (2008), analyzing overall shape as a function of our continuous measure of color, to directly test for a relationship between shape and color, which we expect if body shape and color are correlated (Malek et al., 2012). We first performed a multivariate regression of the Procrustes‐transformed coordinates to calculate a shape score. We then used a mixed model with continuous measures (residual color) and categorical color morph as fixed effects, site as a random effect, centroid size as a covariate, and the regression score representing shape as the outcome variable, followed by Tukey's HSD (with alpha = .05) to find pairwise comparisons. The vectors of regression coefficients from these analyses can be thought of as shape changes per unit of color change. To determine how well each morph is classified by color and shape, we performed a linear discriminant analysis (LDA) in R using the packages “stats” (R Core Team, 2018) and “MASS” (Venables & Ripley, 2002) with categorical color morph (red, black, or mixed) as the grouping factor, and continuous color and the regression shape score as discriminators.

We performed a canonical variate analysis to visualize and statistically assess shape features that best distinguish groups from one another, comparing body shape between color morphs and sexes. We then used a principal component analysis (PCA) to examine variation in shape among males of all color morphs and from all sites. We used PC1 and PC2 of the PCA as outcome variables in linear models to test for variation in shape among categorical color morphs (mixed model, site = random effect) and among sites. To visualize the differences in shape among color morphs, we also performed a PCA for each categorical color morph separately and created wireframe graphs using the independent axes of body shape variation (PC1 and PC2) and compared them to the average shape of all males in MorphoJ.

To assess differences in size among male color morphs, we used a linear mixed model with centroid size as the outcome variable, categorical color morph as a fixed effect, and site as a random effect. Finally, we used a linear mixed model with continuous plate count of both males and females as the outcome variable, categorical color morph as a fixed effect, and site as a random effect to assess differences in lateral plate count among color morphs. Again, we used Tukey's HSD (with alpha = .05) following both mixed models to find pairwise comparisons.

## 3 RESULTS

### 3.1 Water transmittance

We categorized and named sites by the color morph present at a site (e.g., red stickleback are found in red sites). The transmittance of light through water samples at 405 nm varied across red sites (R1 and R2), black sites (B1–B4), the three mixed locations closest to the ocean (M1–M3), and the two mixed locations furthest inland (M4 and M5; *F*_3,45_ = 18.43, *p* < .0001; Figure 3c). The transmittance of light at red sites was higher than that at black sites (Tukey's HSD, *p* < .01) and inland mixed locations (M4 and M5; *p* < .01). Also, the transmittance of light at coastal mixed locations (M1–M3) was higher than that at black sites (*p* < .0001) and inland mixed locations (*p* < .0001). However, transmittance did not differ between red sites and coastal mixed locations (*p* > .05), nor between black sites and inland mixed locations (*p* > .05). This pattern in Connor Creek strongly suggests a gradient in transmittance, wherein water is less tannin‐stained as the creek approaches the ocean.

### 3.2 Male color

**Figure 4:**
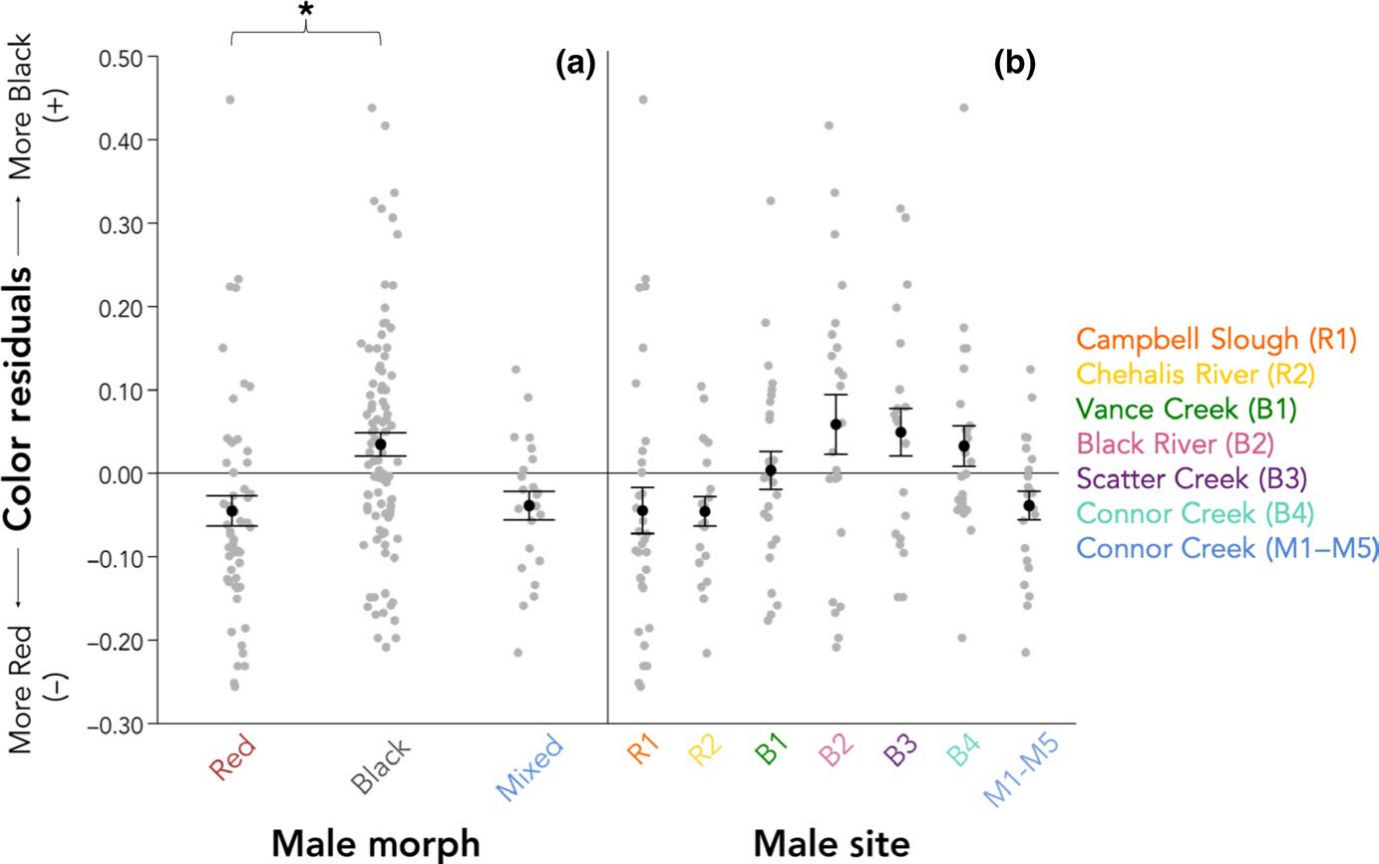
The residual color scores of males differ among morphs and also among sampling sites. (a) Red and black males differ in color, but mixed males do not differ from either. (b) Sites within a morph do not significantly differ in color. Gray points represent raw values, black points and bars represent the least square means from the analysis plus or minus *SE*, and the asterisk indicates a significant difference using Tukey's HSD (*p* ≤ .05)

We found a significant negative regression of black area on red area (*F*_1,170_ = 18.40, *p* < .0001) in addition to quantitative differences in the color of male stickleback commonly called red and black (*F*_2,3.51_ = 10.96, *p* = .031; Figure 4a). Males categorized by researchers (by eye) as “red” were significantly redder than those categorized as “black” (Tukey's HSD, *p* < .05), and black male stickleback were significantly blacker than red stickleback. Males from the five locations within Connor Creek (M1–M5) did not differ in color from one another (*F*_4,18_ = 1.34, *p* = .29) and are hereafter collectively referred to as the “mixed” morph. Mixed males were intermediate in quantitative color and did not differ from either red (*p* > .05) or black males (*p* > .05). We also found overall differences in male color by site (*F*_6,165_ = 3.00, *p* = .0083; Figure 4b), but there were no significant pairwise differences among sites using Tukey's HSD.

### 3.3 Male color and body shape

**Figure 5:**
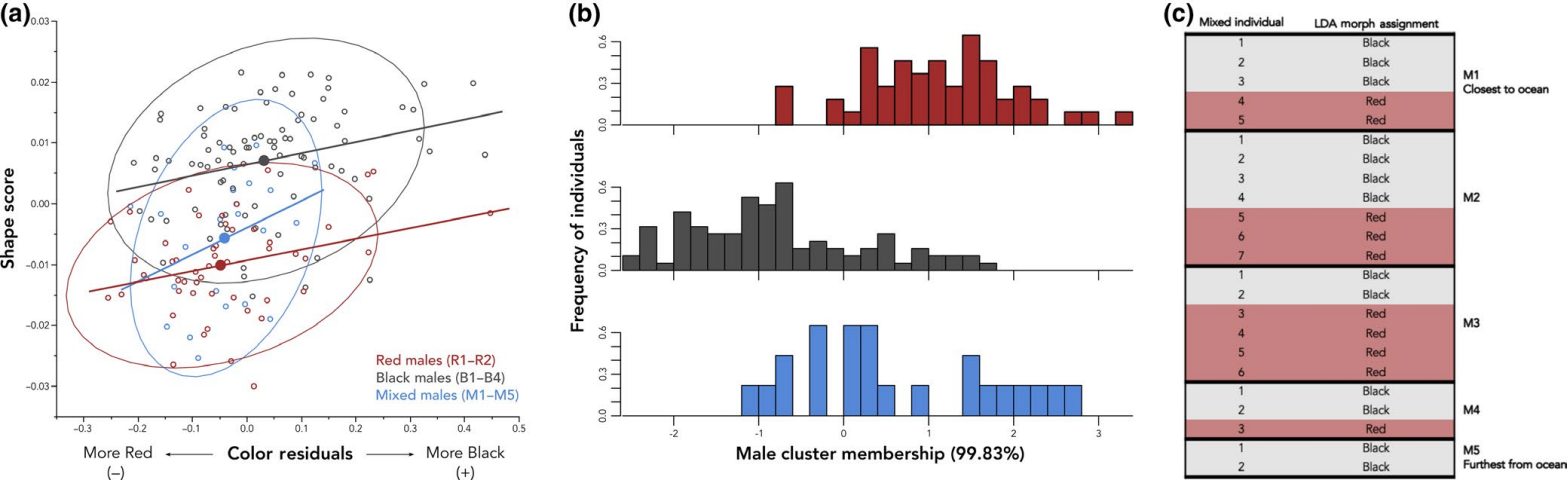
(a) The relationship between male color and body shape. Male residual color and body shape are correlated, but the relationship between residual color and shape scores of males differs among morphs. The relationship between color and shape differs for red and black males, and for black and mixed males, but it does not differ for red males and mixed males. Ellipses represent 95% confidence intervals; closed, colored points indicate the average for each group; and colored lines depict the slope of the interaction for each group. (b) Linear discriminant analysis of males classified by color and shape. Together, male color and shape correctly classify most individuals from allopatric red sites as red and most individuals from allopatric black sites as black. (c) LDA assignments of males belonging to the mixed morph. Nearly half of the individuals from the Connor Creek mixed site (locations M1–M5) are classified as red and half are classified as black (see Table 2 for LDA assignments and proportions)

Overall, we found that color and shape are correlated in this system. There was a significant relationship between continuous color variation and shape variation in male morphs ($\chi_{1}^{2}$ = 13.72, *p* = .0002) that is dependent on categorical color ($\chi_{2}^{2}$ = 23.73, *p* < .0001; Figure 5a). The relationship between color and shape differed between red and black males (Tukey's HSD, *p* < .001), and between black and mixed males (*p* < .001), but the relationship between color and shape did not differ between red and mixed males (*p* = .13). The LDA showed that 99.83% of the discriminability is explained by LD1 (Figure 5b). Of the individuals we categorize as red (sites R1 and R2), 74.1% were classified as red and 25.9% were classified as black by the LDA. Of the individuals we categorize as black (sites B1–B4), 85.3% were classified as black and 14.7% were classified as red by the LDA. No individuals of any morph were classified as mixed by the LDA; however, of the individuals we categorize as mixed (site M1–M5), 43.5% were classified as red and 56.5% were classified as black (Table 2).

**Table 2:** Proportion of stickleback assigned to red, black, or mixed color morphs based on a linear discriminant analysis using color and shape

	LDA Classification		
	Red	Black	Mixed
Red (*N* = 54)	74.1%	25.9%	0%
Black (*N* = 95)	14.7%	85.3%	0%
Mixed (*N* = 23)	43.5%	56.5%	0%

### 3.4 Body shape

**Figure 6:**
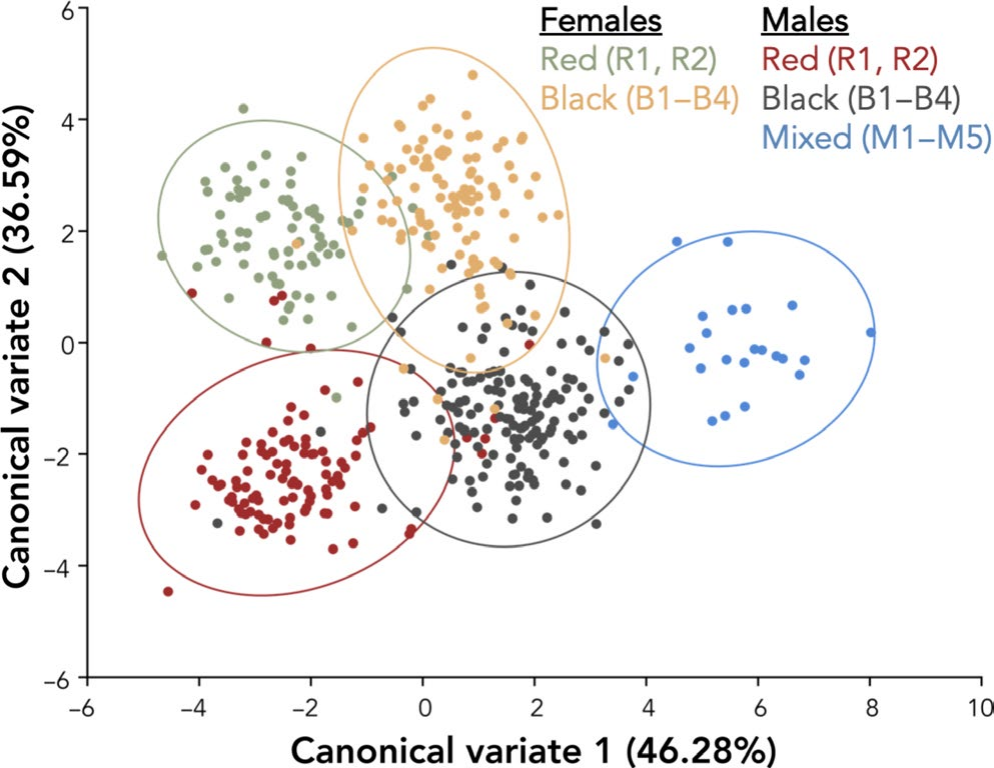
Variation in shape between sexes and color morphs. There is significant variation in shape in all pairwise comparisons of the five groups (see Table 3). Ellipses represent 95% confidence intervals

When fish were placed into five groups by sex and morph (red females, black females, red males, black males, and mixed males), we found significant variation in overall body shape between all groups (Figure 6; Table 3). CV1 explained 46.28% of the total variation in shape and CV2 explained 36.59% of the total variation in shape. Within a color morph, shape significantly differed between the sexes (Procrustes distance_red female − red male_ = 0.053, *p* < .0001; Procrustes distance_black female − black male_ = 0.041, *p* < .0001), and within a sex, shape significantly differed between the morphs (Procrustes distance_red female − black female_ = 0.028, *p* < .0001; Procrustes distance_red male ‐ black male_ = 0.030, *p* < .0001; Procrustes distance_red male − mixed male_ = 0.052, *p* < .0001; Procrustes distance_black male − mixed male_ = 0.036, *p* < .0001).

**Table 3:** Procrustes distances and p‐values comparing shape across sex and color morph

	Black females		Red males		Black males		Mixed males	
	Procrustes distance	*p*‐value	Procrustes distance	*p*‐value	Procrustes distance	*p*‐value	Procrustes distance	*p*‐value
Red females	0.028	<.0001	0.053	<.0001	0.050	<.0001	0.065	<.0001
Black females	‐	‐	0.056	<.0001	0.041	<.0001	0.054	<.0001
Red males	‐	‐	‐	‐	0.030	<.0001	0.052	<.0001
Black males	‐	‐	‐	‐	‐	‐	0.036	<.0001

**Figure 7:**
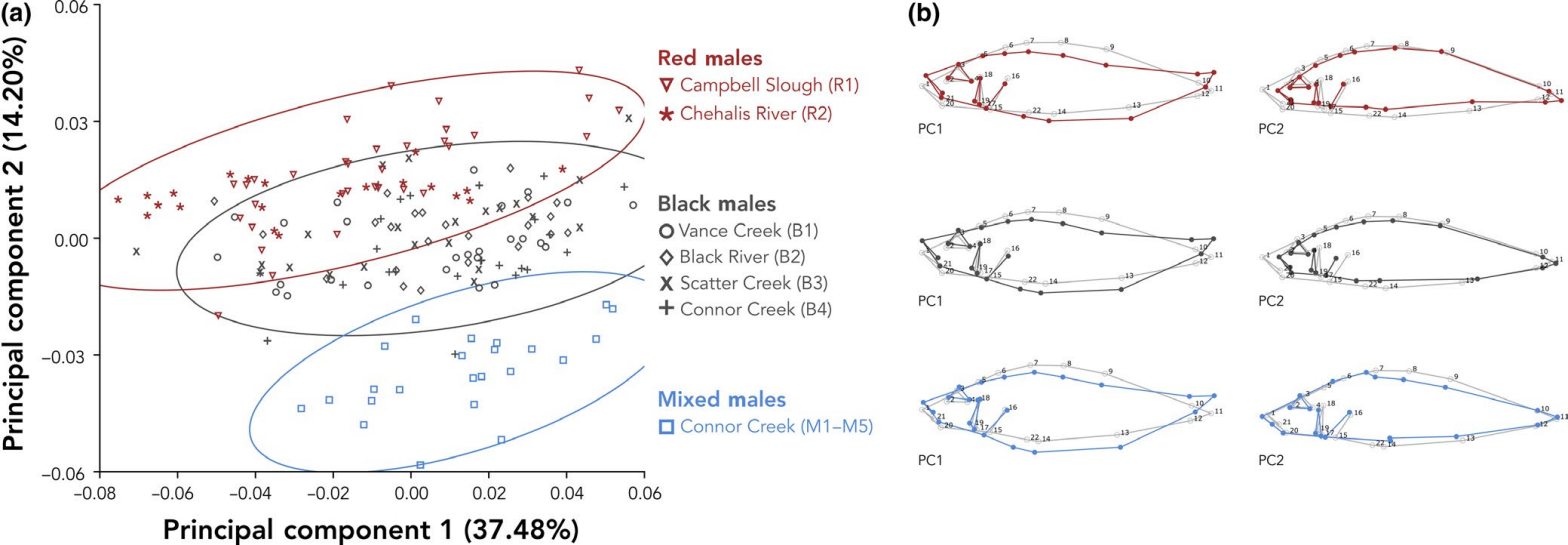
(a) Principal component analysis of shape among all males by color morph and by site. PC1 and PC2 scores differ among morphs and sites (see Tables 4 and 5 for all Tukey's HSD pairwise comparisons). Ellipses represent 95% confidence intervals. (b) Wireframes showing differences in shape between color morphs. In each case, the underlying gray wireframe corresponds to the average of the entire male dataset, and the overlaying colored wireframes show how the shape of males of each color morph differ from the average on PC1 (left) and PC2 (right)

In the principal component analysis investigating shape differences among all males, the major axis of phenotypic variation, PC1, explained 37.48% of the total variation in shape and the second axis of phenotypic variation, PC2, explained 14.20% of the total variation in shape (Figure 7a). The linear model confirmed PC1 (*F*_2,4.12_ = 8.72, *p* = .033) and PC2 (*F*_2,4.00_ = 82.89, *p* = .0006) scores differed among red, black, and mixed male color morphs (Figure 7a; Tukey's HSD pairwise comparisons in Table 4A and B). PC1 (*F*_6,165_ = 5.88, *p* < .0001) and PC2 (*F*_6,165_ = 60.19, *p* < .0001) scores also differed among sites (Figure 7a; Tukey's HSD pairwise comparisons in Table 5A and B). The wireframe graphic of PC1 depicts how each morph deviates in body shape from the average of the entire male dataset whereas PC2 depicts how each morph deviates in face shape from the average of the entire male dataset (Figure 7b).

**Table 4:** Tukey's HSD pairwise comparisons (mean difference ± *SE*) for PC1 scores (A) and PC2 scores (B) of male shape among color morphs. Highlighted cells represent significantly different pairwise comparisons (alpha = .05)

(A)		
Red Males	0.023 ± 0.00063	0.030 ± 0.0090
Black Males	–	0.0071 ± 0.00083

(B)		
Red Males	0.015 ± 0.0027	0.049 ± 0.0038
Black Males	–	0.034 ± 0.0035

**Table 5:** Tukey's HSD pairwise comparisons (mean difference ± *SE*) for PC1 scores (A) and PC2 scores (B) of male shape among sites. Highlighted cells represent significantly different pairwise comparisons (alpha = .05)

(A)						
	R2	B1	B2	B3	B4	M1–M5
R1	0.018 ± 0.0077	0.013 ± 0.0073	0.017 ± 0.0075	0.012 ± 0.0076	0.020 ± 0.0074	0.023 ± 0.0075
R2	–	0.031 ± 0.0081	0.035 ± 0.0084	0.030 ± 0.0085	0.038 ± 0.0083	0.041 ± 0.0084
B1	–	–	0.0037 ± 0.0079	0.0017 ± 0.0080	0.013 ± 0.0073	0.0092 ± 0.0079
B2	–	–	–	0.0054 ± 0.0083	0.0028 ± 0.0081	0.0056 ± 0.0082
B3	–	–	–	–	0.0082 ± 0.0082	0.011 ± 0.0083
B4	–	–	–	–	–	0.0028 ± 0.0081

(B)						
	R2	B1	B2	B3	B4	M1–M5
R1	0.0058 ± 0.0029	0.019 ± 0.0028	0.015 ± 0.0029	0.014 ± 0.0029	0.020 ± 0.0028	0.052 ± 0.0029
R2	–	0.013 ± 0.0031	0.0095 ± 0.0032	0.0086 ± 0.0032	0.014 ± 0.0032	0.046 ± 0.0032
B1	–	–	0.0035 ± 0.0030	0.0044 ± 0.0031	0.0011 ± 0.0030	0.033 ± 0.0030
B2	–	–	–	0.0009 ± 0.0031	0.0046 ± 0.0031	0.036 ± 0.0031
B3	–	–	–	–	0.0055 ± 0.0031	0.037 ± 0.0031
B4	–	–	–	–	–	0.032 ± 0.0031

### 3.5 Body size

**Figure 8:**
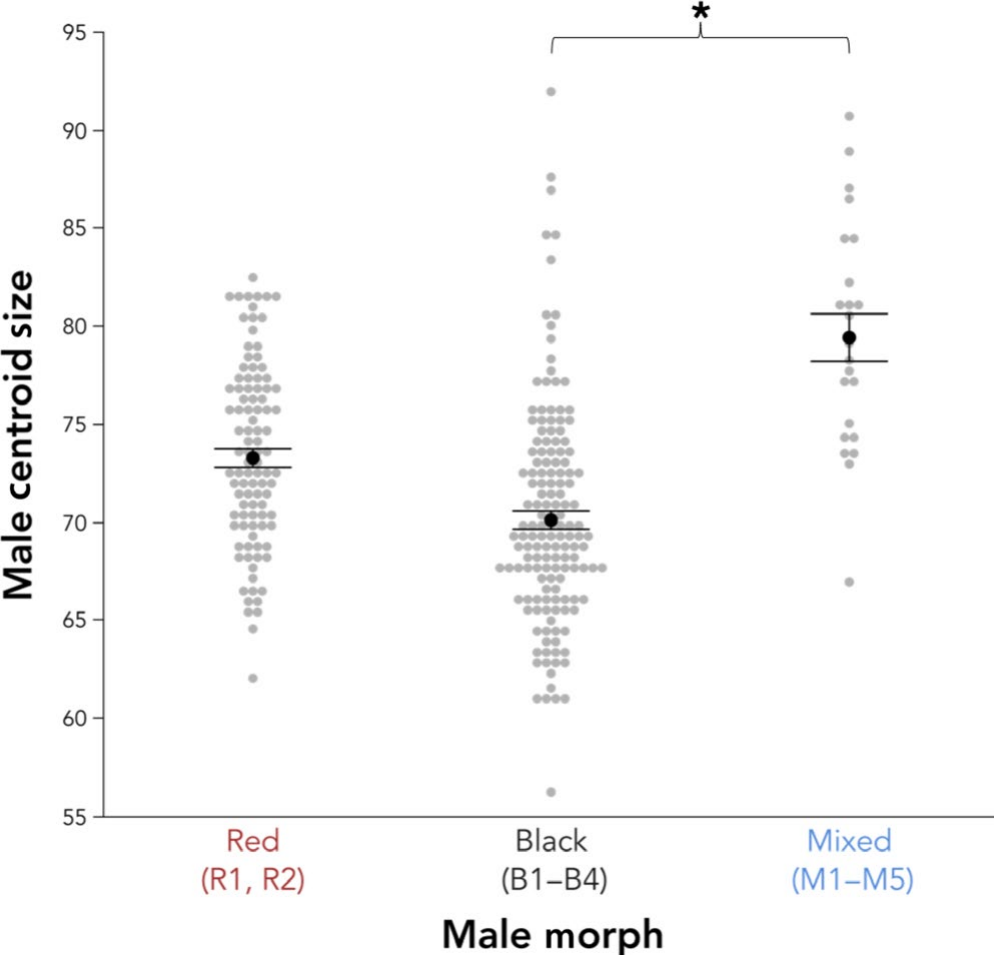
Male size measured as centroid size. Red and mixed males do not differ in size nor do red and black males, but mixed males are larger than black males. Bars represent the least square means from the analysis plus or minus *SE* and the asterisk indicates a significant difference using Tukey's HSD (*p* ≤ .05)

Overall, we found differences in the size of male stickleback of different color morphs (*F*_2,4.69_ = 9.76, *p* = .021; Figure 8). Red males had a centroid size of 73.00 ± 1.26, black males had a centroid size of 70.10 ± 0.91, and mixed males had a centroid size of 79.43 ± 1.94. Red males and black males did not differ in size (Tukey's HSD, *p* > .05), red and mixed males did not differ in size (*p* > .05), but mixed males were significantly larger than black males (*p* < .05).

### 3.6 Lateral plating

**Figure 9:**
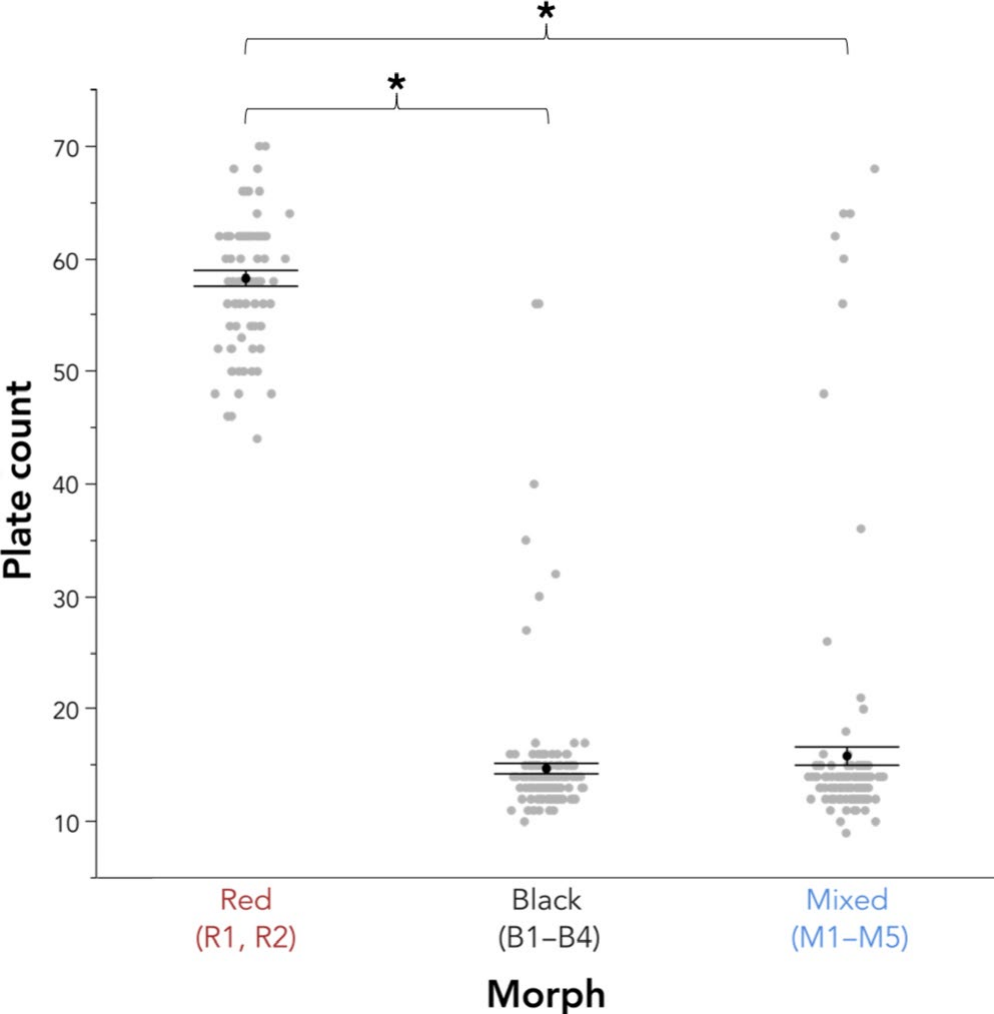
Lateral plate count of both males and females among color morphs. Black and mixed fish do not differ in lateral plate count, but red fish have significantly more body plating than both black fish and more body plating than mixed fish. The gray points represent raw values, the black points and bars represent the least square means from the analysis plus or minus *SE*, and asterisks indicate significant differences using Tukey's HSD (*p* ≤ .05)

We also found differences in lateral body plate count among color morphs (*F*_2,7.47_ = 310.12, *p* < .0001; Figure 9). About 98.8% of red fish were fully plated, and only 1.2% of individuals were partially plated. About 95.8% of black fish were low‐plated, 3.0% were partially plated, and 1.2% were fully plated. About 92.1% of mixed fish were low‐plated, 0.7% were partially plated, 2.6% were intermediately plated, and 4.6% were fully plated (Table 6). All four plate morphs were observed among individuals of the mixed color morph, and the atypical, intermediate plate morph—an uncommon and rare occurrence (Bell et al., 2004)—was only observed among individuals of the mixed color morph. On average, red males had a plate count of 57.61 ± 1.52 plates, black males had a plate count of 14.70 ± 1.07 plates, and mixed males had a plate count of 15.61 ± 1.12 plates. Black and mixed fish did not differ in lateral plate count (Tukey's HSD, *p* > .05), but red fish had significantly more body plating than both black (*p* < .05) and mixed fish (*p* < .05).

**Table 6:** Number of individuals in each color morph categorized by plate morph

	Plate Morph			
	Low	Partial	Intermediate	Full
Red	0	1	0	82
Black	158	5	0	2
Mixed	140	1	4	7

## 4 DISCUSSION

Among freshwater sites in southwest Washington, we found clear phenotypic divergence between red and black stickleback morphs in color, shape, and plating, and evidence consistent with a hypothesis of sensory drive as the prevailing mechanism behind the rapid, evolutionary switch in nuptial coloration in this system. We discovered a region in Connor Creek with a gradient in water color (transmittance at 405 nm) and found that the “mixed” morph has traits that are intermediate to the red and black morphs in some cases and divergent in another case (size). In Connor Creek, variation in water color may play a role in the maintenance of multiple morphs in very close proximity and perhaps contributes to hybridization.

### 4.1 Divergence in color and support for sensory drive

Red coloration in the threespine stickleback is a well‐established component of sexual signaling (McKinnon, 1995; Milinski & Bakker, 1990; Semler, 1971). Black coloration, which is less well‐studied, has been documented in southwest Washington (Hagen & Moodie, 1979; McPhail, 1969; Semler, 1971) and three regions of the Pacific Coast of Canada–Haida Gwaii (Reimchen, 1989), Vancouver Island, and Texada Island (Boughman, 2001). When we assessed color as a continuous variable (the residuals of black color onto red color), we confirmed that red and black stickleback are different from one another and that the mixed morph is intermediate in color to red and black (Figure 4a). Sites within a morph do not vary in color (Figure 4b). Researchers working in this system can easily visually distinguish red stickleback from black stickleback, which are also found in nonoverlapping distribution. The LDA reaffirms our pre‐assigned categories and correctly classified red and black individuals based on their phenotypes (Figure 5b).

When the evolution of black stickleback has been studied, differences in the light environment have been implicated in the switch from red to black male coloration. When there are high concentrations of dissolved organic compounds, such as tannins, in freshwater environments, short wavelength light is filtered out of the visible spectrum, producing a background of red‐shifted light (Reimchen, 1989). The black male sexual signal has high contrast against these tannin‐stained habitats whereas the red male sexual signal has high contrast against the green‐shifted light of most clear, unstained freshwater habitats (Boughman, 2001; Reimchen, 1989; Scott, 2001). Thus, if sensory drive plays a role in this shift in color, we would expect the distribution of color morphs to align with the transmittance properties of their environments. Indeed, we found that the transmittance of light through water at 405 nm was higher in sites where we collected only red stickleback and lower in sites where we collected only black stickleback, indicating that the black morph is found in environments with high tannin staining (Figure 3a).

Interestingly, when we assessed the mixed locations within Connor Creek, we found evidence of an environmental gradient over this short distance. The average transmittance of light through water at the short wavelength end of the spectrum (405 nm) is higher at the three locations closest to the ocean than the two locations furthest inland (Figure 3c). This indicates that water is relatively more clear near the ocean than further inland. In addition to a transition in transmittance properties, we observed that the surrounding habitat also changed along Connor Creek from areas with high vegetation and deeper water (B4) to sand dunes and shallower water (M1) as the creek approached the Pacific Ocean (Figure 1b). At the time of sampling, we did not anticipate the degree to which stickleback nesting habitat would vary along Connor Creek. Future work will address habitat variation more systematically to elucidate the role of environment in the transmittance of light and phenotypic divergence of stickleback across southwest Washington. It would be fruitful to assess characteristics like surrounding vegetation, salinity, water depth, stream order and flow, tidal range, substrate composition, nest locations, diet, and predator composition as well (Marques, Lucek, et al., 2017). This is particularly important as the use of certain sampling methods (e.g., minnow traps) limits our understanding of abiotic and biotic microhabitat differences among morphs and collection sites (Marques, Lucek, et al., 2017).

Given that our water transmittance data suggest that this has been an important contributor to the evolution of black nuptial coloration, we expected that stickleback in Connor Creek would be more phenotypically similar to the red morph in locations closest to the ocean, where water is relatively clear and unstained, and gradually shift to an appearance more phenotypically similar to the black morph in the locations furthest inland, where water is red‐shifted and tannin‐rich. Instead, we found that nearly half of the males we categorized as belonging to the mixed morph were classified as red and half were classified as black by the LDA and that they were distributed almost evenly throughout the first four mixed locations (i.e., red‐like fish were not only found in locations closer to the ocean and black‐like fish were not only found in locations furthest inland; Figure 5c; Table 2). It is possible that the lack of such a phenotypic cline is due to the migration patterns of stickleback and the dramatic habitat variation we observe in Connor Creek. Given that freshwater stickleback can travel up to five kilometers to breeding sites, anadromous migrants can travel large distances of at least 10 kilometers to freshwater breeding sites (Snyder & Dingle, 1989), and the breeding season of freshwater and anadromous stickleback overlap (Bell & Foster, 1994), individuals may be freely interbreeding along the creek, preventing the establishment of a measurable gradient across a short geographical range.

### 4.2 Divergence in shape and size

Body shape and size are well‐studied components of sexual signaling in the marine‐benthic and benthic‐limnetic stickleback species complexes and have been shown to vary both between sexes (Cooper et al., 2011) and between morphs in several populations (Albert et al., 2007; Head et al., 2013; Malek et al., 2012; Taylor et al., 2006). However, little is known about how shape and size diverge between morphs that inhabit different freshwater river or stream habitats. Here, we found variation in shape of stickleback from streams in southwest Washington between sexes and color morphs (Figure 6; Table 3). Among only males, body shape differs between red and black morphs and sites (Figure 7a; Table 4A and B), but size does not (Figure 8). Mixed males differed in shape from both red and black males on one axis of a PCA (PC2; Figure 7a, Table 4B) and by site (Figure 7a; Table 5B). As depicted in the wireframe graphics, PC1 appears to best explain variation in body depth and shape, whereas PC2 appears to best explain variation in face structure and shape (Figure 7b). Thus, mixed males differ from red and black males primarily in face shape. Additionally, mixed males were also larger than black males but did not differ in size from red males (Figure 8). In the benthic‐limnetic pair, differences in body shape arose by adaptation to local foraging and predator environments (reviewed in McKinnon & Rundle, 2002). In our red‐black color morphs, it is possible that divergent natural selection has first led to divergence in shape from anadromous ancestors as the fish adapted to freshwater environments (McPhail, 1994), which was followed by divergence in shape of the morphs through adaptation to specialized and different freshwater niches.

Alternatively, animals often examine more than one signal simultaneously when assessing competitors (Candolin & Voigt, 2001) and potential mates (Candolin, 2000). Evolutionary changes in one sexually selected trait may be correlated with changes in others and are simultaneously under sexual selection in this system. When traits are correlated, through pleiotropy or linkage disequilibrium, direct selection on one may consequently lead to indirect selection on an associated trait (Brooks & Endler, 2001). Malek et al. (2012) found that markers associated with male color were significantly associated with body shape in a quantitative trait locus analysis of benthic and limnetic stickleback in Enos Lake, motivating our assessment of relationships between color and shape in SW Washington stickleback. We found that residual color and shape are indeed correlated and that this relationship differs among color morphs (Figure 5a). The relationship between color and shape differs for red and black males, and for black and mixed males, but it does not differ for red and mixed males. In addition to expressing preferences for extensive red coloration, there is also evidence that female stickleback have preferences for male body shape in some groups (Head et al., 2013). Male color and shape may thus be correlated through simultaneous, direct natural selection on both traits during adaptation to freshwater environments, due to sexual selection driven by female preference for both traits, or through indirect selection of one as a byproduct of direct selection on the other. Ultimately, the relationship between male color and shape suggests that when one is favored by natural or sexual selection, we might expect the other to evolve in concert if genetic correlations are persistent.

### 4.3 Variation and surprises in lateral body plating

While the overwhelming majority of fish from red sites were fully plated, black and mixed fish were predominately low‐plated with few partial, intermediate, and full morphs (Table 6; Figure 9). The occurrence of fully plated individuals in red sites is unusual, in that we expect a loss or reduction in body armor following invasions from oceanic to freshwater environments (Hagen & Gilbertson, 1973; Bell & Foster, 1994). However, fully plated populations have been documented in this region before (Hagen & Gilbertson, 1973). The presence of fully plated stickleback in Washington rivers could indicate that natural selection has favored the maintenance or reappearance of extra lateral plates in certain habitats (Kitano et al., 2008; Reimchen, Bergstrom, & Nosil, 2013). Alternatively, fully plated red fish may live in environments subject to more or different predators than low‐plated black fish or could be recently introduced marine stickleback, either through the migration of anadromous populations or through anthropogenic transfer from coastal to freshwater sites (Adachi et al., 2012; Currey et al., 2019).

Though the number of plates did not differ between mixed and black morphs, it is interesting to note the unexpected presence of the intermediately plated individuals within the mixed morph, which to our knowledge, has not before been documented in this region. In Loburg Lake, Alaska, Bell et al. (2004) also discovered rare intermediately plated individuals and suggested that this plate morph does not occur in older polymorphic populations and is likely the result of novel allele combinations generated during adaptive radiation. This leads us to hypothesize that intermediately plated stickleback in Connor Creek may result from recent hybridization.

### 4.4 Accumulation of evidence for Connor Creek as a potential hybrid zone

We have established that a suite of traits differs between the red and black stickleback morphs in SW Washington. However, the mixed morph differs from red and black males in some, but not all, traits investigated. To review, we discovered that males from the mixed morph are intermediate in color relative to red and black males, and display a range of color values that overlap with and are not different than either type statistically (Figure 4a). The transmittance of light at 405 nm of the three mixed locations closest to the ocean (M1–M3) does not differ from the transmittance at red sites and the transmittance of the two locations furthest inland (M4 and M5) does not differ from the transmittance at black sites (Figure 3c), though transmittance is higher (clearer water) at the coastal locations than the inland locations (Figure 3c), similar to the higher transmittance at red sites than at black sites. We also observed that there are dramatic changes in habitat within Connor Creek (Figure 1b). However, we must thoroughly investigate these ecological characteristics to understand if and how they contribute to phenotypic divergence. Further, male color and shape are correlated, and this relationship differs between black and mixed males, but not between red and mixed males (Figure 5a). Within the mixed morph, an LDA based on shape and color classified slightly more individuals in Connor Creek as “black” than “red” (Figure 5b).

When assessing only shape, mixed males do not differ from red and black morphs in body shape (PC1) but do differ from both morphs in head shape (PC2), which is evident from the larger and more elongated head (Figure 7b). Mixed males were larger than black males (Figure 8), had fewer lateral plates than red fish, but did not differ in lateral plating when compared to black fish (Figure 9). However, 4.6% of the sampled individuals were fully plated, which we otherwise saw only at red freshwater sites and is also characteristic of the anadromous form (Bell, 2001).

Together, the intermediate coloration, the variation in shape patterns, the increased size, and polymorphic plating relative to the red and black morphs all create a unique and perplexing story within Connor Creek. Although we are not certain how much of the measured variation in morphology and color reflects underlying genetic variation, many of the traits we examined are shown to be heritable (Aguirre, Doherty, & Bell, 2004; McPhail, 1977) and have been genetically mapped (Albert et al., 2007; Colosimo et al., 2005; Cresko et al., 2004; Peichel & Marques, 2017; Schluter et al., 2004; Yong et al., 2016). Given the genetic basis of these traits, the larger size of anadromous stickleback relative to freshwater forms (Head et al., 2013), the similarity in nuptial coloration and body armor of the red freshwater morph and anadromous form (Bell, 2001; McKinnon & Rundle, 2002), as well as its proximity to the Pacific Ocean, it is possible that the phenotypic variation we observe in Connor Creek is the result of introgressive hybridization between anadromous stickleback and the black morph that resides further upstream, or a combination of introgression and environmental variation. Given how frequently marine and freshwater environments come into contact, it is not surprising that hybrid zones between freshwater‐resident and anadromous stickleback are widespread (Hagen, 1967; Hendry, Bolnick, Berner, & Peichel, 2009; Jones, Brown, Pemberton, & Braithwaite, 2006; McPhail, 1994).

By determining the extent to which the traits we investigated here are under ecological and/or sexual selection, future work will illuminate how natural selection and sexual selection may interact to drive, maintain, or limit divergence among morphs in SW Washington. Recent population genomic studies have begun to unravel the evolutionary forces that underly phenotypic change following the colonization of new habitats (Hoekstra et al., 2004; Jones, Grabherr, et al., 2012; Marques, Taylor, et al., 2017; Rosenblum et al., 2004). It would be interesting to know whether the phenotypic divergence that we observe in color, shape, size, and plating between freshwater morphs is associated with genetic divergence among populations and color morphs. Additionally, including pure anadromous and marine populations from this region and assessing genetic variation alongside phenotypic change will allow us to address our hypothesis of an anadromous‐freshwater hybrid zone in Connor Creek. This will ultimately contribute to our growing understanding of how biodiversity is shaped under strong evolutionary forces and over short time scales.

## CONFLICT OF INTEREST

The authors have no conflicts of interest to declare.

## AUTHOR CONTRIBUTIONS

CSJ, WRL, and RMT conceived and designed the study; CSJ, WRL, BTK, LFS, and ANS contributed to phenotypic data collection; CSJ and WRL performed all data analyses and interpretation; CSJ and RMT drafted the paper; and all authors contributed to revising the manuscript and gave their final approval for publication.

## Data Availability

Morphological and light transmittance data are publicly available at the Dryad repository: https://doi.org/10.5061/dryad.dr7sqv9vd.
